# Structural Models for Cytochrome P450—Mediated Catalysis

**DOI:** 10.1100/tsw.2003.41

**Published:** 2003-06-20

**Authors:** David F.V. Lewis

**Affiliations:** School of Biomedical and Life Sciences, University of Surrey, Guildford, Surrey, UK

**Keywords:** cytochrome P450, structural modeling, quantitative structure-activity relationships

## Abstract

This review focuses on the structural models for cytochrome P450 that are improving our knowledge and understanding of the P450 catalytic cycle, and the way in which substrates bind to the enzyme leading to catalytic conversion and subsequent formation of mono-oxygenated metabolites. Various stages in the P450 reaction cycle have now been investigated using X-ray crystallography and electronic structure calculations, whereas homology modelling of mammalian P450s is currently revealing important aspects of pharmaceutical and other xenobiotic metabolism mediated by P450 involvement. These features are explored in the current review on P450-based catalysis, which emphasises the importance of structural modelling to our understanding of this enzyme's function. In addition, the results of various QSAR analyses on series of chemicals, which are metabolised via P450 enzymes, are presented such that the importance of electronic and other structural factors in explaining variations in rates of metabolism can be appreciated.

## INTRODUCTION

The cytochromes P450 (CYP) constitute a superfamily of over 1200 heme-thiolate enzymes which are responsible for the Phase I metabolism of many drugs and other foreign compounds, although some P450s have important endogenous roles in, for example, the biosynthesis of steroid hormones[1,2]. It is generally accepted that over 90% of drug oxidations in man are mediated by P450 enzymes, and it would appear that the major catalysts of human drug metabolism are CYP3A4, CYP2D6, and CYP2C subfamily enzymes[3]. Of these, CYP3A4-mediated catalysis accounts for over 34% of drug oxidations, whereas CYP2D6 is involved in around 19% of drug oxidations in man with enzymes of the CYP2C subfamily accounting for almost 16% (CYP2C8 and CYP2C9) and with over 8% for CYP2C19 involvement. Enzymes of the CYP1A subfamily also play a significant role in drug metabolism, especially CYP1A2 (8%). Consequently, there is considerable current interest in the P450 superfamily of mono-oxygenases, especially with respect to drug metabolism in man. First of all, it may be helpful to consider the catalytic mechanism of substrate oxygenation mediated via P450 before leading into a discussion of quantitative relationships between structure and rates of metabolism.

## P450-Mediated Catalysis

The P450 catalytic cycle (Fig. 1) involves substrate binding to the enzyme's active site, which brings about displacement of bound water molecules, and this desolvation process is associated with a perturbation of heme iron spin-state equilibrium in favour of the high-spin ferric iron (reviewed in [4]). The accompanying conformational change in the P450 protein brings about favourable binding with its redox partner, and the lowering of P450 redox potential (i.e., becoming less negative) facilitates electron transfer from the reductase molecule (reviewed in [5]). Following reduction of the heme iron from Fe(III) to Fe(II), oxygen binding occurs via ligation of the sixth ligand site such that the iron returns to the low-spin state.

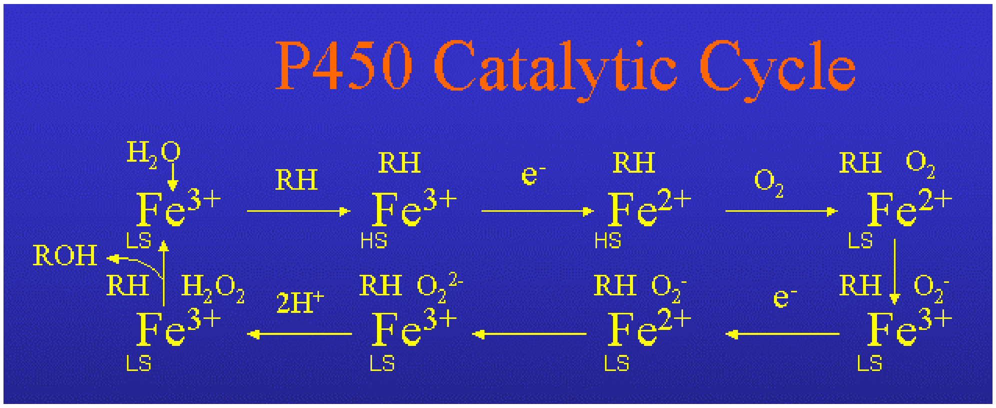

**FIGURE 1.** The P450 catalytic cycle showing various stages in the overall process for oxygenation of a typical substrate[4]. HS = high spin; LS = low spin; RH = substrate; ROH = metabolite.

There is spectroscopic evidence for the presence of superoxide at this stage, which suggests that the Fe(II)$O_2$ state is in equilibrum with the more reactive form represented by Fe(III)$O_2^-$, and a further electron transfer process then reduces the ferric iron back to ferrous while maintaining the electronic state of the bound oxygen species. The ferrous superoxide complex, possibly in equilibrium with the ferric peroxide state, represents a highly reactive oxygenating species for achieving single oxygen insertion into the bound substrate following scission of the dioxygen bond. Although there is experimental evidence for an iron-bound peroxide[6,7] in some P450-mediated reactions, it is possible that an iron-oxene intermediate could act directly in single oxygen insertion into the substrate, following cleavage of the dioxygen bond (reviewed in [4]). The full mechanism of oxygen insertion into substrates[8,9] is an area of considerable interest and debate, with hard experimental evidence being difficult to obtain because of the rapid rate of this final stage in the P450 catalytic cycle[10,11]. However, the formation of an active oxygen intermediate comprised of a single oxygen atom bound to the heme iron of P450 represents a likely possibility for enabling direct oxygen insertion into the majority of known P450 substrates (reviewed in [4]) although mechanistic schemes involving peroxy species can also be formulated in several examples (reviewed in [12]).

The general equation for P450-mediated oxygenation of a typical substrate (RH) to form a hydroxy metabolite (ROH) is as follows:

$$RH + O_2 \xrightarrow[2H^+, 2e^-]{P450} ROH + H_2O$$

Where the two reducing equivalents ($2H^+$, $2e^-$) are supplied sequentially via the mediation of a redox partner such as an iron-sulphur redoxin or an FAD- and FMN-containing oxidoreductase flavoprotein (reviewed in [5]). In most bacterial systems, the redox partner utilized is an iron-sulphur redoxin, which employs an FAD-dependent reductase to transfer reducing equivalents from NADH. A similar situation exists in the mitochondrial P450 pathway, although the reductant is NADPH in this case. For microsomal P450s, however, the redox partner involved is an oxidoreductase flavoprotein that transfers reducing equivalents from NADPH. In addition, there is also evidence for the role of cytochrome $b_5$ in mediating the second electron transfer pathway in liver microsomal P450 systems (reviewed in [5]).

## Quantitative Structure-Activity Relationships (QSARs) in P450 Catalysis

Table 1 presents a summary of quantitative structure-activity relationship (QSAR) studies for seven series of compounds for which rate or clearance data are available. These are categorized as substrates of CYP2B (toluenes, barbitals, and dimethylanilines), CYP2E (halothanes and nitriles), CYP3A (benzodiazepines and sodium channel blockers), and CYP4A (carboxylic acids). It can be appreciated from an inspection of Table 1 that different factors appear to be involved in QSAR evaluations for the various series of chemicals, although there are some recurring features such as frontier orbital energies and lipophilicity, as measured be either log P or log $D_{7.4}$ (which is the ionisation-corrected lipophilicity at pH7.4).

As far as frontier orbitals are concerned, the LUMO and HOMO energies are embodied in the $\Delta E$ parameter, where $\Delta E = E_{LUMO} - E_{HOMO}$, and this quality has also been termed the “hardness” of the molecule. Moreover, ionization potential (IP) is essentially the same as $E_{HOMO}$, albeit of opposite sign, and this features in Eq. 3a and 5a, Table 1. For substrates of CYP2B enzymes, correlating factors appear to depend on the nature of the series investigated although dipole moment is important in QSARs for both toluenes and dimethylanilines (Eq. 1a and 7b, respectively). In addition, there is a role for desolvation of the CYP2B active site accompanying substrate binding for barbital drugs as the clearance ratio is determined by a quadratic expression in log P, the lipophilicity parameter (Eq. 2a, Table 1). However, it is possible that transport across the endoplasmic reticular membrane is also indicated by this parabolic relationship in log P, as it is known that similar quadratic curves in log P relate to cellular transport[13].

For the CYP2E substrates, it is found that the ionization potential (equivalent to - $E_{HOMO}$) correlates with the rate of metabolism for halothanes, and this quantity is also encountered in other studies on rates of P450-mediated reactions (reviewed in [4,14]). However, for a large group of nitriles, it is found that a combination of $\Delta E$ and molecular diameter correlates well ($R = 0.92$) with rate of metabolism attributable to CYP2E involvement (see Table 1, Eq. 4a). Consequently, in this example, the molecular size is important for binding to the CYP2E active site. Moreover, the “hardness” factor is also apparent in this correlation, and this is the energy gap between HOMO and LUMO frontier orbitals[15].

Substrates of CYP3A tend to be relatively large molecules, in contrast with those of CYP2E that are generally of low molecular weight[16]. For a series of benzodiazepines and sodium channel blockers, there is a good correlation ($R = 0.91$) between intrinsic clearance (Cl) and a combination of ionization potential (IP) and log $D_{7.4}$, which is the lipophilicity factor corrected

**TABLE 1**
**QSARs for Rates of P450-Mediated Reactions[17,18]**

| | n | s | R | F |
|---|---|---|---|---|
| 1. Toluenes (CYP2B substrates)<br>a) $\log k_{cat} = 0.024\ \text{vol} - 0.227\mu - 1.143$<br>$(\pm 0.005)$ | 8 | 0.1067 | 0.98 | 52.70 |
| 2. Barbitals (CYP2B substrates)<br>a) $\log Cl_{ratio} = 2.250 \log P - 0.300 \log P^2 - 4.100$<br>$(\pm 0.188)$ $(\pm 0.035)$ | 10 | 0.1085 | 0.99 | 461.42 |
| 3. Halothanes (CYP2E substrates)<br>a) $\log \text{Rate} = 26.899 - 2.577\ \text{IP}$<br>$(\pm 0.21)$ | 6 | 0.1270 | 0.99 | 145.20 |
| 4. Nitriles (CYP2E substrates)<br>a) $\log \text{EtOH/Glucose} = 0.894 - 0.111\ \text{diam} - 0.07\ \Delta E$<br>$(\pm 0.109)$ $(\pm 0.004)$ | 18 | 0.0313 | 0.92 | 42.10 |
| 5. Benzodiazepines and $Na^+$ channel blockers (CYP3A substrates)<br>a) $\log Cl = 0.643 \log D_{7.4} - 0.981\ \text{IP} + 9.334$<br>$(\pm 0.109)$ $(\pm 0.300)$ | 12 | 0.4978 | 0.91 | 21.39 |
| 6. Carboxylic acids (CYP4A substrates)<br>a) $k_\omega = 33.145 - 1.63\ N_C$<br>$(\pm 0.06)$ | 4 | 0.3820 | 0.999 | 369.37 |
| 7. N, N-Dimethylanilines (CYP2B substrates)<br>a) $\log k_{cat} = 0.691\ E_L + 0.638$<br>$(\pm 0.076)$ | 9 | 0.1010 | 0.96 | 83.67 |
| b) $\log k_{cat} = 1.331 - 0.147\ \mu$<br>$(\pm 0.010)$ | 9 | 0.0668 | 0.98 | 200.39 |

*Note*: n = number of observations; s = standard error; R = correlation coefficient; F = variance ratio, $k_{cat}$ = rate constant for P450-mediated catalysis; vol = molecular volume; $\mu$ = dipole moment; Cl = metabolic clearance; IP = ionization potential; EtOH/glucose = rate ratio of CYP2E-mediated related to control; diam = molecular diameter; $D_{7.4}$ = distribution coefficient at pH7.4; $k_\omega$ = rate constant for ω-hydroxylation; $N_c$ = number of carbon atoms in molecule; $E_L$ = LUMO energy.

for acid/base dissociation at pH 7.4 (see Eq. 5, Table 1). It is interesting to note that IP is a factor for both CYP2E and CYP3A substrates, indicating that this parameter is likely to be involved in rates of P450-mediated metabolism irrespective of the molecular size of substrate. However, in the case of the two series of CYP3A substrates mentioned above, it is apparent that the distribution coefficient ($\log D_{7.4}$) is an essential component in describing the variation in clearance for these compounds.

For a small series of long-chain fatty acids that are able to act as CYP4A substrates, it is observed that the rate constant for ω-hydroxylation is directly proportional (R = 0.99) to the number of carbon atoms in the chain, as shown by Eq. 6a, Table 1. This finding is consistent with active site modelling of fatty acid substrates within the CYP4A subfamily[19] although it is clear that a larger dataset of substrates should be investigated to ascertain the significance of this observation.

It is apparent, therefore, that the rates of P450-mediated reactions and metabolic clearance of substrates can be studied using QSAR techniques, and parameters largely governing the variation in rates have been generally established, although there is scope for further work in this area (reviewed in [18]).

## Structurally Characterised P450 Enzymes

To date, only a relatively small number of P450 structures have been resolved crystallographically. Most of these are from bacterial sources, although there has been progress recently in the characterisation of eukaryotic P450s. Bacterial P450 crystal structures[20,21,22,23,24] include those of CYP101, CYP102, CYP107, and CYP108, whereas eukaryotic P450s that have had their structures determined via X-ray crystallography include CYP51, CYP55, and CYP2C5; the latter being a mammalian form of the enzyme. CYP51 and CYP55 represent fungal forms of the enzyme that are associated with lanosterol 14α-demethylase and nitric oxide reductase, respectively. There is current interest in the CYP2C5 and CYP51 crystal structures[25] due to the fact that they represent useful templates for modelling human P450s involved with drug metabolism and steroidogenesis, respectively. However, the unusual I helix conformation in CYP51 probably limits the utility of this structure as a template for P450s other than those of the CYP51 family, although homology modelling of aromatase (CYP19) may be a possibility due to a similarity between their catalytic mechanisms.

By and large, however, the tertiary fold is generally well conserved in structurally characterised P450s, as are the secondary structural elements (α-helical and β-sheet domains). This is particularly apparent when the bacterial P450 structures are compared[26] but even the mammalian form, CYP2C5, bears similarities with the bacterial type II P450, CYP102[27]. A number of these structures contain bound substrates or inhibitors (i.e., CYP101, CYP102, CYP51, and CYP107) and it is apparent that there is complementarity between groupings on the substrate and active site amino acid side-chains in the vicinity of the heme moiety. Consequently, key amino acid residues in the P450 active sites seem to be involved in orientating the relevant substrates for metabolism at the experimentally observed positions. For example, tyrosine-96 in CYP101 is involved in hydrogen bonding to the ketonic oxygen of the camphor substrate that, together with a number of hydrophobic contacts, orientates the molecule for 5-exo-hydroxylation. In fact, it is possible to use substrate-binding contact information to estimate the likely binding energy of the enzyme-substrate interaction[28,29].

Table 2 summarizes the currently available information on structurally characterised P450s and their redox partners, from which it can be appreciated that many of the redox partners in various P450 systems from different classes (i.e., bacterial and microsomal systems) have been investigated using either X-ray crystallography or NMR spectroscopy (reviewed in [2]). For example, the bacterial $P450_{cam}$ system from *Pseudomonas putida* involving the enzyme itself and its redox partner, putidaredoxin, have both been structurally characterised via these techniques (see Table 2 for details). Furthermore, there is increasing information about mammalian microsomal P450 systems, where the oxidoreductase and cytochrome $b_5$ redox components have both been characterised in addition to the rabbit microsomal P450, CYP2C5. Additionally, the Class II prokaryotic P450 system from *Bacillus megaterium* has also been investigated using X-ray crystallography, particularly for the enzyme itself (i.e., $P450_{BM3}$ or CYP102) and its complex with the FMN domain of oxidoreductase (see Table 2 for details).

It is apparent that relatively well-defined regions of the P450 enzymes are associated with redox partner interactions (reviewed in [5]) where complementary pairing of oppositely charged

**TABLE 2**
**Structurally Characterised P450s[a]**

| CYP | Description | Iron State | PDB Code | Resolution (Å) | References |
|---|---|---|---|---|---|
| 101 | Camphor-bound $P450_{cam}$ | $Fe^{3+}_{HS}$ | 2cpp | 1.63 | 20 |
| 101 | $P450_{cam}$:CO complex[b] | $Fe^{2+}_{LS}$ | 3cpp | 1.90 | 30 |
| 101 | Substrate-free $P450_{cam}$[c] | $Fe^{3+}_{LS}$ | 1phc | 2.20 | 31 |
| 101 | Adamantane-bound $P450_{cam}$[d] | $Fe^{3+}_{HS}$ | 4cpp | 2.10 | 32 |
| 101 | 5-hydroxycamphor-bound $P450_{cam}$ | $Fe^{3+}_{LS}$ | 1noo | 2.00 | Protein Databank, 1995[e] |
| 102 | Substrate-free $P450_{BM3}$ | $Fe^{3+}_{LS}$ | 2hpd | 2.00 | 21 |
| 102 | Substrate-free $P450_{BM3}$ | $Fe^{3+}_{LS}$ | 2bmh | 2.00 | 33 |
| 102 | Palmitoleate-bound $P450_{BM3}$ | $Fe^{3+}_{HS}$ | 1fag | 2.70 | 24 |
| 102 | Substrate-free $P450_{BM3}$:T268A | $Fe^{3+}_{LS}$ | 1fah | 2.30 | 34 |
| 102 | FMN and heme domains | $Fe^{3+}_{LS}$ | 1bvy | 2.03 | 35 |
| 107 | Erythronolide-bound $p450_{eryF}$ | $Fe^{3+}_{HS}$ | 1oxa | 2.10 | 22 |
| 108 | Substrate-free $P450_{terp}$ | $Fe^{3+}_{LS}$ | 1cpt | 2.30 | 23 |
| 55 | Substrate-free $P450_{nor}$ | $Fe^{3+}_{LS}$ | 1rom | 2.00 | 36 |
| 2C5 | Substrate-free P450 2C5 | $Fe^{3+}_{LS}$ | 1dt6 | 3.00 | 27 |

LS = low-spin; HS = high-spin.

[a] A total of 32 individual bacterial P450 structures are accessible via the Protein Databank of which 22 are of $P450_{cam}$, 6 are of $P450_{BM3}$, 2 are of $P450_{nor}$, 1 is of $P450_{terp}$, and 1 of $P450_{eryF}$.
[b] Camphor moves about 1Å away from the heme relative to that in the enzyme-substrate complex.
[c] A water molecule occupies the 6th ligand distal to the heme, and there are 6 water molecules in the active site previously occupied by camphor in the 2cpp structure.
[d] Other substrates cited in this reference include: adamantanone, camphene, and norcamphor.
[e] Deposited by T.L. Poulos.

amino acid residues play an important role in binding of the redox partner complex. By and large, it is found that basic residues on the P450 enzyme form ion-paired electrostatic interactions with acidic residues on the redox partner, be it either a redoxin, reductase or cytochrome $b_5$[5].

## HOMOLOGY MODELS OF P450 ENZYMES

It is possible to use the crystal structures of P450s for the modelling of other forms of the enzyme based on primary sequence homology[1,2]. This procedure has been employed in the construction of a significant number of P450s, especially the mammalian microsomal forms associated with Phase 1 metabolism of drugs and other xenobiotics[29]. Essentially, this involves the production of a satisfactory alignment between the template and target sequences, followed by changing the relevant amino acid residues from the template crystal structure to those required in the target P450. Then, the raw model is energy minimised using molecular mechanics to give rise to a geometry-optimised structure of the P450 in question. For P450s within the CYP2 family, there is a relatively high primary sequence homology (50–80%) with the mammalian P450 CYP2C5, which is of known 3-D structure[27]. Consequently, this relatively new crystal structure constitutes the preferred template for the homology modelling of human P450s within the CYP2 family[29]. Furthermore, it is possible to construct models of CYP1 family enzymes from the CYP2C5 crystallographic template that show consistency with experimental information in the form of site-directed mutagenesis data and positions of substrate metabolism. This is also found to be the case for the CYP2 family models with respect to the human orthologues[29]. However, as far as CYP3A4 is concerned, this major human enzyme involved in the Phase 1 oxidation of many drug substrates, some of which also exhibit allosteric effects[37], bears closer similarity (in terms of primary sequence identity) with CYP102 rather than CYP2C5, although the identities are relatively close (27% for CYP102 and 24% for CYP2C5). Consequently, this may represent an example of a mammalian enzyme where the CYP2C5 crystallographic template may not necessarily represent the most appropriate paradigm for homology modelling. Nevertheless, it is reasonable to assume that the CYP2C5 structure is likely to be an appropriate starting point for constructing models of most human P450s that are involved in drug metabolism, such as those from families CYP1 and CYP2. Fig. 2 shows an example of a human drug-metabolising P450 enzyme (namely, CYP3A4) with a selective inhibitor, ketoconazole, present in the putative active site region. There are favourable interactions with complementary amino acid residues lining the active site, which appear to orientate the molecule such that the heme-ligating azole ring lies directly above the heme iron, and at a distance that is consistent with iron-nitrogen coordinate bonding. This mode of interaction is supported by the appearance of a Type II spectral change accompanying ketoconazole binding to CYP3A4, and the contacts with complementary amino acid residues within the enzyme's putative active site are in agreement with experimental evidence from site-directed mutagenesis studies (reviewed in [2]).

In conclusion, therefore, it appears that crystal structures of P450 enzymes can be employed as templates for the construction of 3-D models for human forms of the enzyme, and the results of such modelling exercises are in harmony with the body of experimental evidence which support the technique[2,29]. Consequently, it is feasible to utilize such models for the prescreening of novel development compounds that are destined for human exposure. In addition, there are biotechnological possibilities in using the P450 system[38] especially where P450 mimics can be employed as novel catalysts[39].

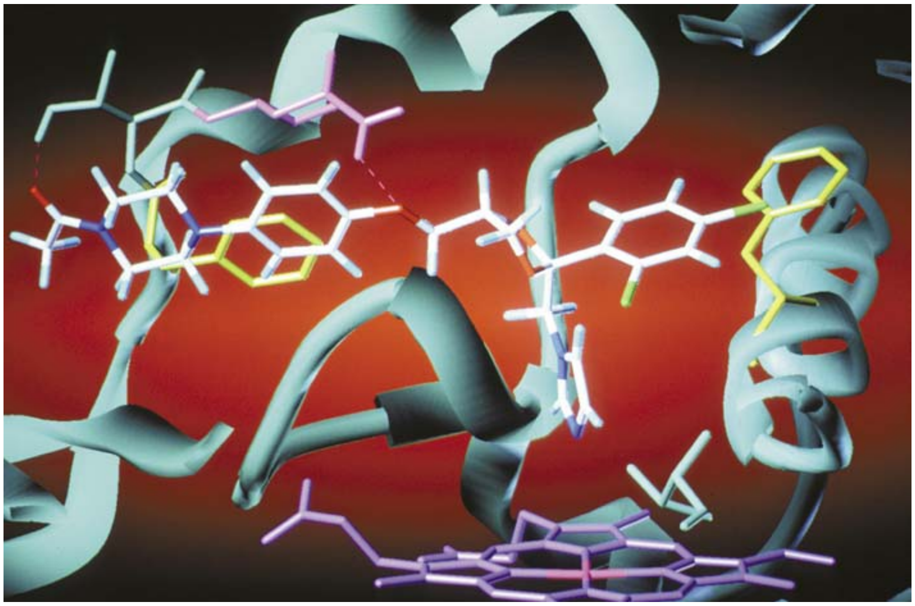

**FIGURE 2.** The putative active site of CYP3A4 showing the selective inhibitor, ketoconazole, orientated for heme ligation via a combination of hydrogen bonding (dashed lines) and π-π stacking with complementary aromatic residues (shown in yellow). Ketoconazole is coloured by atom type (carbon - white, hydrogen - pale blue, nitrogen - blue, oxygen - red, chlorine - green), whereas the amino acid residues are coloured by type (amides in magenta; aromatic residues in yellow; hydroxyl sidechains in pale green; hydrophobic residues in pale blue; acidic residues in red; basic residues in blue). The heme moiety is shown in purple and the iron atom is at its centre.

## ACKNOWLEDGEMENTS

The financial support of GlaxoSmithKline Research and Development Limited, Merck Sharp and Dohme Limited, and the University of Surrey Foundation Fund is gratefully acknowledged.
